# Effect of different cardioprotective methods on extracorporeal circulation in fetal sheep: a randomized controlled trial

**DOI:** 10.1186/s13019-021-01486-y

**Published:** 2021-04-17

**Authors:** Yi-bo Yan, Shuo Shi, Qian-biao Wu, Jin-sheng Cai, Bin-feng Lei

**Affiliations:** 1 Department of Cardiac Surgery, The First Affiliated Hospital of Guangxi Medical University, Nanning, Guangxi Zhuang Autonomous Region People’s Republic of China; 2 Department of Thoracic Surgery, The First Affiliated Hospital of Guangxi Medical University, Nanning, Guangxi Zhuang Autonomous Region People’s Republic of China

**Keywords:** Apoptosis, Congenital heart disease, Creatine kinase–muscle band, Extracorporeal circulation, Fetal sheep, HTK preservation solution (Custodiol®), St Thomas’ Hospital cardioplegic solution, Troponin

## Abstract

**Background:**

Congenital heart disease is a leading cause of death in newborns and infants. The feasibility of fetal cardiac surgery is linked to extracorporeal circulation (ECC); therefore, cardioplegic solutions need to be effective and long-lasting.

**Methods:**

Eighteen pregnant sheep were divided into an ECC-only group, St. Thomas’ Hospital cardioplegic solution (STH1) group (STH group), and HTK preservation solution (Custodiol®) group (HTK group). Markers of myocardial injury including troponin I (cTnI), troponin T (cTnT) and creatine kinase myocardial band (CKMB) were measured at specific time points (T1: pre-ECC, T2: 30 min of ECC, T3: 60 min of ECC, T4: 60 min post-ECC, T5: 120 min post-ECC). Myocardial tissue was removed from the fetal sheep at T5, and apoptosis was detected by TUNEL staining.

**Results:**

Changes in the serum cTnI, cTnT and CKMB concentrations were not significantly different among the three groups before and during the ECC(T1,T2,T3). At 60 min after ECC shutdown(T4), cTnI and cTnT concentrations were significantly higher in the STH group than before the start of ECC. The concentration of cTnI was higher in the STH group than in the HTK and ECC-only groups. The concentration of cTnT was higher in the STH group than in the ECC-only group. At 120 min after ECC shutdown(T5), cTnI and cTnT concentrations were significantly higher in the ECC and HTK groups than before the start of ECC, and CKMB concentration was significantly higher in STH and HTK groups. The concentrations of cTnT, cTnI and CKMB was higher in the STH group than in the HTK and ECC-only groups. The number of TUNEL-positive cells in the HTK and STH groups was higher than in the ECC-only group. The number of TUNEL-positive cells in the STH group was higher than in the HTK group. There was no statistically significant difference among the groups in the heart rate and mean arterial pressure after ECC.

**Conclusion:**

The HTK preservation solution was significantly better than STH1 in reducing the release of cardiomyocyte injury markers and the number of apoptotic cells in fetal sheep ECC. Fetal sheep receiving ECC-only had an advantage in all indicators, which suggests ECC-only fetal heart surgery is feasible.

## Background

Congenital heart disease is one of the leading causes of death in newborns and infants and accounts for about 1% of all congenital malformations [1]. Although there have been impressive advances in pediatric cardiac surgery in the last 20 years, some complex cardiac malformations (left ventricular dysplasia syndrome, pulmonary atresia, etc.) still result in high intrauterine death rates, postnatal mortality, and complications [2–4].

The early fetal heart pattern is formed by the eighth week in utero, and further development of the heart is influenced by the pattern of fetal blood flow [5, 6]. Theoretically, if congenital heart disease can be operated on during the fetal period, the transformation of an abnormality into a complex one can be prevented, complex postnatal cardiac surgery can be avoided, the heart can be given a chance to develop further in utero, and secondary morphologic damage can be avoided [7–9].

The introduction of minimally invasive and robotic techniques in cardiac surgery, the development of fetal anesthesia concepts, advances in ECC technology and improvement in ECC equipment have made fetal cardiac surgery possible. Nevertheless, concerns regarding myocardial protection have been a major hindrance, and the perioperative myocardial injury and postoperative cardiac dysfunction are yet to be addressed [10]. Because of the fetus’s unique characteristics, the need for the protection of immature fetal myocardium is much higher than that in the adults. There are significant differences between the immature and mature myocardium in terms of function, ultrastructure, energy metabolism, and tolerance of ischemia and hypoxia. In order to meet the high level of fetal surgical refinement required, cardioplegic solutions need to be effective and prolonged. At present, the protection of immature myocardium is still being explored [11]. Research on the theory of fetal ECC began in 1984, with attempts at ECC in fetal sheep first reported by Slate et al. at the University of California [12]. Based on many experiments on fetal sheep and a thorough understanding of fetal sheep physiology, the technology for ECC in fetal sheeps is largely mature [13].

In this study, we used a small centrifugal pump to establish an extracorporeal circulation model in fetal sheep. We used three ECC methods. The first one is to keep the heart from arresting, which means the hearts are not arrested but just maintained on ECC. The second one is the use of St Thomas’ Hospital cardioplegic solution (STH1). The third one is the use of HTK preservation solution (Custodiol®). We monitored fetal cardiac function and compared the effects of the three methods on fetal sheep, including cardiomyocyte apoptosis and levels of markers of myocardial injury during and after the surgery, given the clinical applicability of this approach.

## Materials and methods

### Study design (Fig. 1)

Eighteen pregnant sheeps (100–120 days gestation, 150 days at term) weighing 25.72 ± 4.29 kg (17.5–32 kg), with 18 fetuses, were used for the study. We divided the fetal sheep into three groups of six: the ECC-only group, the STH group, and the HTK. In the ECC-only group, we established ECC for the fetal sheep for 60 min, then arrested it and observed the sheep for 120 min. In the STH and HTK groups, the ascending aorta of the fetal sheep was occluded, and the heart was perfused with St Thomas’ Hospital cardioplegic solution (STH1) and HTK preservation solution (Custodiol®) to arrest the fetal heart for 30 min. The fetal aorta was then reopened to allow both, body and extracorporeal circulation, and after the heart had successfully resumed beating for 30 min, the ECC was stopped, and the sheep were observed for 120 min.

**Fig. 1:**
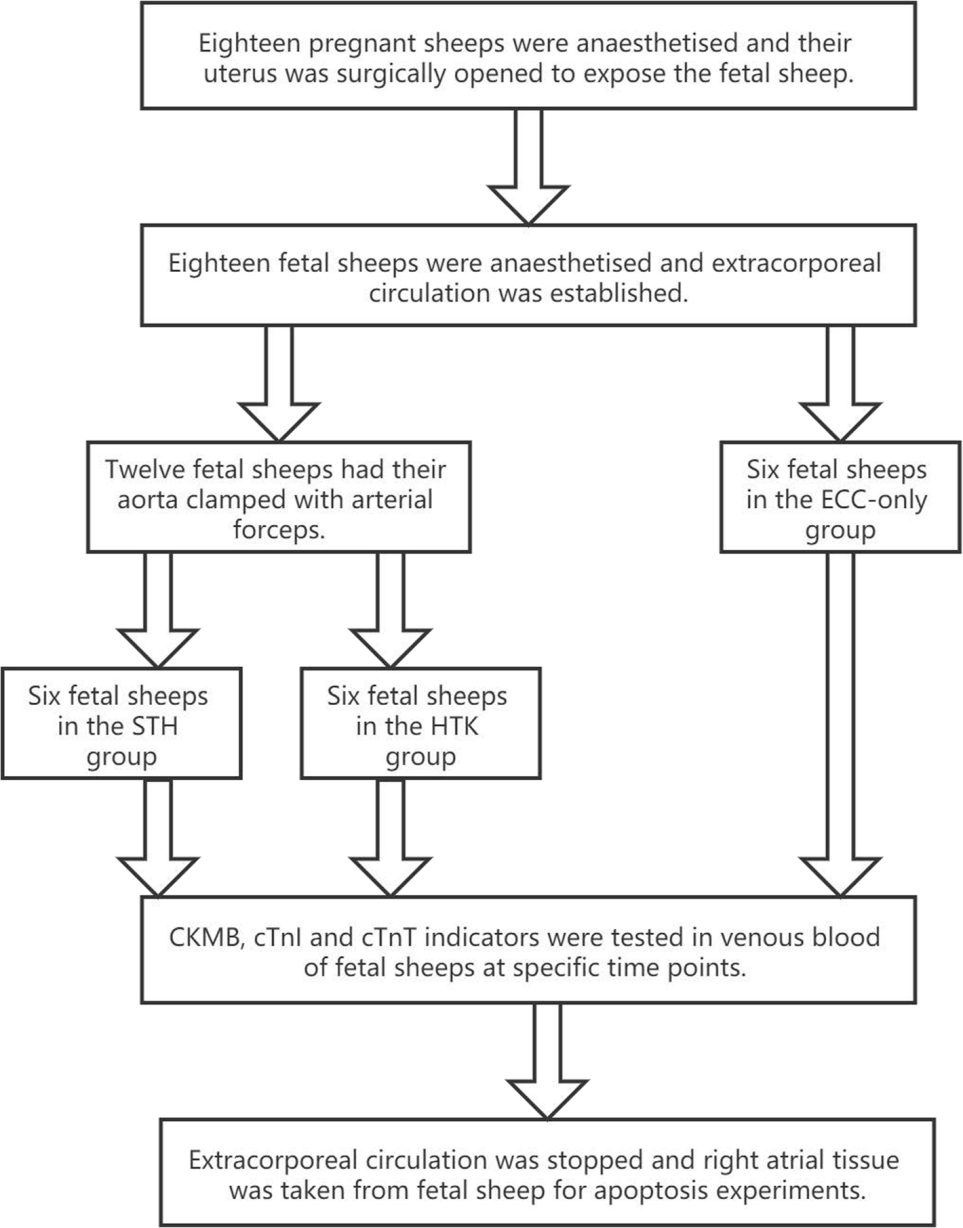
Study design

### Preoperative preparation and anesthesia

Pregnant sheeps were fasted for 24 h. Preoperatively, we prepared the skin of the abdomen, back, and neck of the pregnant sheep and administered a preoperative intramuscular injection of atropine 0.5–1.0 mg/kg and ketamine 10–20 mg/kg into the femoral area. After the sheep’s conjunctival reflex disappeared, the sheep was lifted onto the operating table, and electrodes were attached to its back. The trachea was intubated, and the sheep were ventilated mechanically with a tidal volume of 10–15 mL/kg and an inhalation oxygen concentration of 40–50%, at a frequency of 15–20 times/minute. A cardiac monitor was attached to the forelimb of the pregnant sheep. The femoral artery was punctured and intubated, and an arterial tester was connected to monitor arterial blood pressure. The major surgical equipment and drugs used in the surgeries are presented in Table 1.

**Table 1:** Major surgical equipment and drugs

Name	Company	Specification
Extracorporeal Circulator	STOCKERT,Inc.GER	Shiley
Variable temperature water tank	STOCKERT,Inc.GER	16S093
Multi-parameter monitor	GE,Inc.GER	DragerInfinity Delt
Anesthesia machine	BOCGroup,Inc.USA	EXCEL 210 ANS
Fentanyl Citrate Injection	HUMANWELL, CHN	10 mL:0.5 mg
Ketamine hydrochloride injection	HUMANWELL, CHN	2mmL:0.1 g
Atropine hydrochloride injection	HUMANWELL, CHN	1 mL:0.5 g
Vecuronium Bromide for Injection	HARBIN MEDISAN PHARMACEUTICAL CO., LTD	4 mg
HTK preservation solution (Custodiol®)	Dr. Franz Koehler Chemie GmnH	1000 mL
St Thomas’ Hospital cardioplegic solution (STH1)	Institute of Cardiovascular Diseases, Guangxi Medical University	1000 mL

### Surgical procedures and extracorporeal circulation

To ensure the survival of the fetal sheeps, we perform the surgery in a warm animal operating room at 28 °C. The skin of the pregnant sheep’s lower abdomen was incised, and the uterus was surrounded with gauze pads moistened with warm saline for support. Anesthetic drugs (fentanyl 25–50 μg/kg and vecuronium bromide 0.1–0.2 mg/kg) were injected directly through the uterine wall into the muscles of the fetus’s hind limbs, selecting a site without placental vessels. After the fetal movement disappeared, a 4–6 cm incision was made through the uterine wall in the area without placental blood vessels. Then, the amniotic cavity was opened, the fetal chest exposed, and one upper limb was gently exposed. The axillary artery was exposed and punctured for the placement of a tube to allow monitoring of the fetal arterial blood pressure and heart rate. A mid-thoracic incision was made on the fetal sheep, and the superficial tissue was incised. The space around the aorta and pulmonary artery was dissected.

In the ECC-only group, we established ECC for the fetal sheep for 60 min, then stopped it and observed the sheep for 120 min. In the STH and HTK groups, the ascending aorta of the fetal sheep was occluded, and the heart was perfused with HTK preservation solution (Table 2) and STH1 (Table 3) to arrest the fetal heart for 30 min. When perfusing the hearts of fetal sheep in the STH and HTK groups, we considered that a lower temperature cardioplegic solution (4 °C) might influence the body temperature of the fetal sheep, so we perfused at a slower rate of 30 mL/min, with 50–100 mL per perfusion until the heart arrested. The fetal aorta was then reopened to allow both, body and ECC; after the heart had successfully resumed beating for 30 min, the ECC was stopped, and the sheep were observed for 120 min. The fetal sheep ECC flow rate was above 200 mL/kg/min, and the water tank temperature was kept constant at 40 °C. The axillary artery was exposed and a tube was punctured to allow monitoring of fetal arterial blood pressure and heart rate.

**Table 2:** HTK preservation solution (Custodiol®). Composition. 1000 mL perfusion solution contains

0.8766 g	sodium chloride	15.0 mmol
0.6710 g	potassium chloride	9.0 mmol
0.8132 g	magnesium chloride·6H_2_O	4.0 mmol
3.7733 g	histidine hydrochloride·2H_2_O	18.0 mmol
27.9289 g	histidine	180.0 mmol
0.4085 g	tryptophan	2.0 mmol
5.4651 g	mannitol	30.0 mmol
0.0022 g	calcium chloride·2H_2_O	0.015 mmol
0.1842 g	potassium hydrogen 2-oxopantendioate	1.0 mmol

**Table 3:** St Thomas’ Hospital cardioplegic solution (STH1). Composition. 1000 mL perfusion solution contains

8.4240 g	sodium chloride	144.0 mmol
1.4900 g	potassium chloride	20.0 mmol
1.5200 g	magnesium chloride	16.0 mmol
0.2664 g	calcium chloride	2.4 mmol
0.2728 g	procaine hydrochloride	1.0 mmol

The ECC-only group was maintained on the ECC for 60 min and then arrested for 120 min. After 120 min, 5 mL of 10% potassium chloride was injected intravenously into the fetal sheep, and the heartbeat was arrested rapidly. For convenience and efficiency, right atrial tissue was collected from the fetal sheep and stored for subsequent experiments [14]. The fetal sheep were removed and detached from the ECC, and the uterus and abdominal incision of the ewe were sutured. When the ewes awoke from anesthesia, they were returned to the breeding center to continue rearing.

### Collection and preservation of specimens

The venous blood of fetal sheep was collected at various time points and centrifuged at 1500 g at 4 °C for 15 min. The supernatant was extracted and transferred to the Eppendorf tubes (approximately 0.5 mL per tube) and stored at −80 °C in a freezer for further testing. The concentrations of troponin T (cTnT), troponin I (cTnI), and creatine kinase–muscle band (CKMB) in the supernatant were determined by the enzyme-linked immunosorbent assay at five different time points: T1 (before the start of ECC); T2 (30 min of ECC); T3 (60 min of ECC); T4 (60 min after ECC shutdown) and T5 (120 min after ECC shutdown).

A piece of myocardial tissue of the right atrium of the fetus, of size 1–2 cm, was cut out and placed in a test tube containing 4% formalin solution, sealed, and kept at room temperature. The tissue was dehydrated and embedded in the paraffin wax for slicing. Cells were uniformly stained with TUNEL to facilitate the observation of apoptosis under a 400 × optical microscope; 10 non-overlapping fields of view were randomly selected, and TUNEL-stained cells counted. The apoptosis of cardiomyocytes was detected by combining the morphological characteristics and staining of the apoptotic cells. Apoptotic cells were stained brown with the TUNEL stain and exhibited chromatin concentration and fragmentation, DNA marginalization, and the lysis of the nuclear membrane. The apoptotic index was calculated as follows:

$$ \mathrm{Apoptosis}\ \mathrm{index}\ \left(\mathrm{AI}\right)=\mathrm{apoptotic}\ \mathrm{cell}\ \mathrm{number}/\left(\mathrm{number}\ \mathrm{of}\ \mathrm{apoptotic}\ \mathrm{cell}\mathrm{s}+\mathrm{number}\ \mathrm{of}\ \mathrm{normal}\ \mathrm{cell}\mathrm{s}\right)\times 100\%. $$

### Statistical methods

All data collected in this study were processed using SPSS v.21.0 statistical software (IMB SPSS, Armonk, NY), and the results were expressed as mean ± standard deviation (x ± s). Significant differences between two groups were tested using Student’s *t*-test, and comparisons between multiple groups were made using one-way analysis of variance (*P* < 0.05). One-way ANOVA set at *P* < 0.05 followed by POST HOC LSD (*P* < 0.05) was applied to evaluate differences in myocardial injury marker levels and the number of TUNEL-positive cells between groups at specific time points. *P* < 0. 05 was considered statistically significant.

## Results

### Preoperative characteristics

Twenty-two pregnant sheeps with 22 fetuses were used to develop the ECC direct intracardiac surgery model. Two of the pregnant sheep died of respiratory depression and cardiac arrest during the induction of anesthesia. The remaining 20 ewes were successfully revived after removing the fetuses and were returned to the breeding center. One fetal sheep was intubated into the main pulmonary artery between the outer and middle membranes, which was not detected in time, and it died of cardiac arrest after the start of ECC. Another fetal sheep died due to the failure of cardiac resuscitation, so the experiment was completed with 18 fetal sheeps.

There were no statistically significant differences in the gestational age and body weight between the three groups (Table 4). The STH group had less cardioplegic solutions perfusion than the HTK group (*P* < 0.05), and the STH group required less time to return to a normal heart rate than the HTK group (*P* < 0.05) (Table 4).

**Table 4:** Comparison of general information and intraoperative condition of the three groups of fetal sheep ($\overline{\mathrm{x}}$ ± s)

	ECC-only group (*n* = 6)	STH group (*n* = 6)	HTK group (*n* = 6)
Age (d)	123.71 ± l1.98	122.52 ± 12.33	130.10 ± 01.23
Weight (kg)	1.21 ± 0.56	1.24 ± 0.47	1.33 ± 0.70
cardioplegic solutions perfusion (mL/kg)	–	47.0 ± 8.4	58.0 ± 0.0*
Time to return to normal heart rate (s)	–	45.0 ± 4.3	61.0 ± 3.5*

*P < 0.05, comparison with STH group

### Perioperative hemodynamics

The changes in the heart rate and blood pressure in the three groups of fetal sheep between the T1, T4, and T5 points were not statistically different among the groups. At T2, the STH and HTK groups showed higher ECC flow rate than the ECC-only group (*P* < 0.05). Heart rates were lower in the STH and HTK groups at T3 than at T1 (*P* < 0.05), and the STH group had lower heart rates than the HTK group (*P* < 0.05). The heart rate at T4 was lower than the basal values at T1 in the STH and HTK groups (*P* < 0.05), and that in the STH group was lower than that in the HTK group (*P* < 0.05). At T3, the mean arterial pressure in the STH group was lower than the basal value at T1 point (*P* < 0.05) and was lower than that in the HTK group (*P* < 0.05). There was no statistically significant difference among the groups in the heart rate and mean arterial pressure at T4 and T5 (Table 5).

**Table 5:** Comparison of perioperative hemodynamics in three groups of fetal sheep ($\overline{\mathrm{x}}$ ± s)

	T1	T2	T3	T4	T5
Heart rates (bpm)					
ECC-only group	173.1 ± 20.10	169.1 ± 17.18	171.7 ± 22.21	171.5 ± 18.58	178.1 ± 10.12
STH Group	175.0 ± 15.12	–	110.3 ± 22.10*	177.5 ± 17.14	173.2 ± 19.56
HTK Group	174.6 ± 21.00	–	151.1 ± 19.99*#	174.2 ± 15.11	177.8 ± 11.16
Average Arterial Pressure (mmHg)					
ECC-only group	57.1 ± 15.67	57.3 ± 17.42	56.9 ± 19.11	57.1 ± 15.12	56.5 ± 18.36
STH Group	56.9 ± 13.81	57.5 ± 13.55	50.0 ± 16.11*	59.5 ± 17.22	57.1 ± 16.39
HTK Group	56.9 ± 16.85	56.6 ± 17.34	58.0 ± 19.73#	58.2 ± 16.55	56.8 ± 15.93
ECC flow rate (mL/kg/min)					
ECC-only group	–	101.5 ± 29.22	–	–	–
STH Group	–	114.9 ± 28.45‡	–	–	–
HTK Group	–	113.3 ± 29.76‡	–	–	–

*EC* Extracorporeal circulation

T1: Before start of ECC.

T2: 30 min of ECC.

T3: 60 min of ECC.

T4: 60 min after ECC shutdown.

T5: 120 min after ECC shutdown.

* *P* < 0.05, compared with the pre-ECC; #*P* < 0.05, compared with the STH group; ‡ *P* < 0.05, compared with the ECC-only group

### Indicators of myocardial impairment at various time points in different groups of fetal sheep

There were no significant differences in the serum concentrations of cTnT, cTnI, and CKMB in fetal sheep before the start of ECC between the three groups (Tables 6, 7, 8).

**Table 6:** Changes in the concentration of cTnI in the three groups. ($\overline{\mathrm{x}}$ ± s)

Groups	T1	T2	T3	T4	T5
ECC-only group	59.03 ± 3.21	58.23 ± 4.21	60.74 ± 2.31	65.74 ± 3.25#	73.43 ± 3.37*#
STH Group	60.21 ± 3.23	60.38 ± 5.27	61.70 ± 4.51	87.52 ± 4.25* ‡	90.14 ± 5.96* ‡
HTK Group	59.01 ± 2.19	61.27 ± 3.43	65.69 ± 3.24	67.17 ± 4.56#	83.50 ± 4.63* ‡ #

*ECC* Extracorporeal circulation

T1: Before start of ECC.

T2: 30 min of ECC.

T3: 60 min of ECC.

T4: 60 min after ECC shutdown.

T5: 120 min after ECC shutdown.

* *P* < 0.05, compared with the pre-ECC; #*P* < 0.05, compared with the STH group; ‡ *P* < 0.05, compared with the ECC-only group

**Table 7:** Changes in the concentration of cTnT in the three groups ($\overline{\mathrm{x}}$± s)

Groups	T1	T2	T3	T4	T5
ECC-only group	101.83 ± 12.54	100.11 ± 10.31	104.27 ± 12.11	105.44 ± 9.40 #	119.85 ± 11.07*#
STH Group	103.57 ± 16.32	103.49 ± 10.12	105.96 ± 17.28	117.31 ± 7.75*‡	136.41 ± 9.02*‡
HTK Group	99.40 ± 13.53	103.78 ± 14.56	107.93 ± 10.85	110.62 ± 9.54	129.69 ± 8.90*#

*ECC* Extracorporeal circulation

T1: Before start of ECC.

T2: 30 min of ECC.

T3: 60 min of ECC.

T4: 60 min after ECC shutdown.

T5: 120 min after ECC shutdown.

* *P* < 0.05, compared with the pre-ECC basis; # *P* < 0.05, compared with the STH group; ‡ *P* < 0.05, compared with the ECC-only group

**Table 8:** Changes in the concentration of CKMB in the three groups ($\overline{\mathrm{x}}$± s)

Groups	T1	T2	T3	T4	T5
ECC-only group	0.51 ± 0.01	0.51 ± 0.02	0.55 ± 0.01	0.56 ± 0.01	0.59 ± 0.02
STH Group	0.50 ± 0.04	0.50 ± 0.08	0.54 ± 0.08	0.55 ± 0.02	0.66 ± 0.03* ‡
HTK Group	0.53 ± 0.01	0.53 ± 0.04	0.53 ± 0.07	0.54 ± 0.03	0.64 ± 0.06* ‡ #

*ECC* Extracorporeal circulation

T1: Before start of ECC.

T2: 30 min of ECC.

T3: 60 min of ECC.

T4: 60 min after ECC shutdown.

T5: 120 min after ECC shutdown.

* *P* < 0.05, compared with the pre-ECC basis; # *P* < 0.05, compared with the STH group; ‡ *P* < 0.05, compared with the ECC-only group

### Changes in the concentration of cTnI

**Fig. 2:**
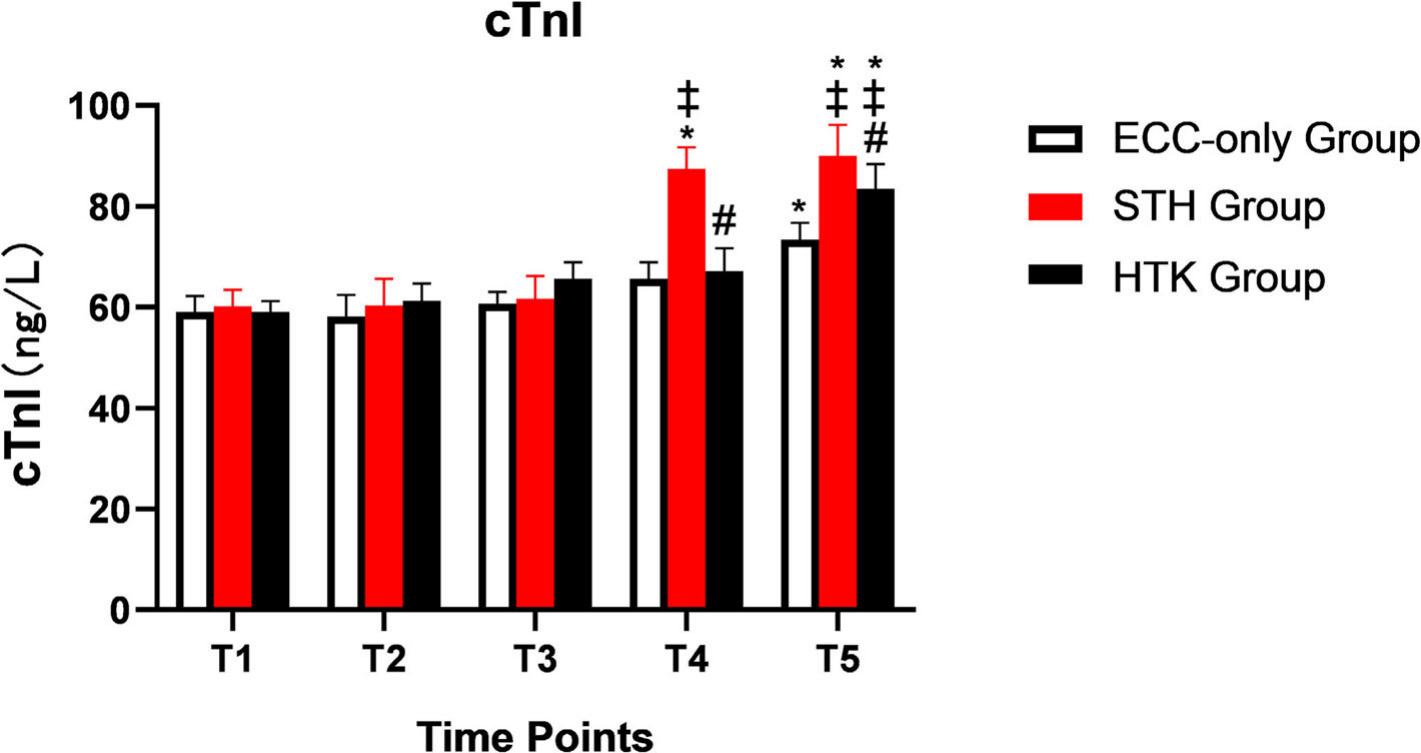
The concentration of cTnI in the three different groups. Note: * *P* < 0.05, compared with the pre-ECC basis; # *P* < 0.05, compared with the STH group; ‡ *P* < 0.05, compared with the ECC-only group

There was no statistically significant difference in cTnI among the three groups at T1, T2, and T3. The cTnI level in the STH group at T4 was significantly higher than that before the start of ECC (*P* < 0.05). The cTnI level in the HTK and ECC-only groups at T5 was significantly higher than that before the start of ECC (*P* < 0.05). The concentration of cTnI was higher in the HTK group than in the ECC-only group at T5 (*P* < 0.05). The concentration of cTnI was higher in the STH group than in the HTK and ECC-only groups at T4 and T5 (*P* < 0.05) (Table 6 and Fig. 2).

### Changes in the concentration of cTnT

**Fig. 3:**
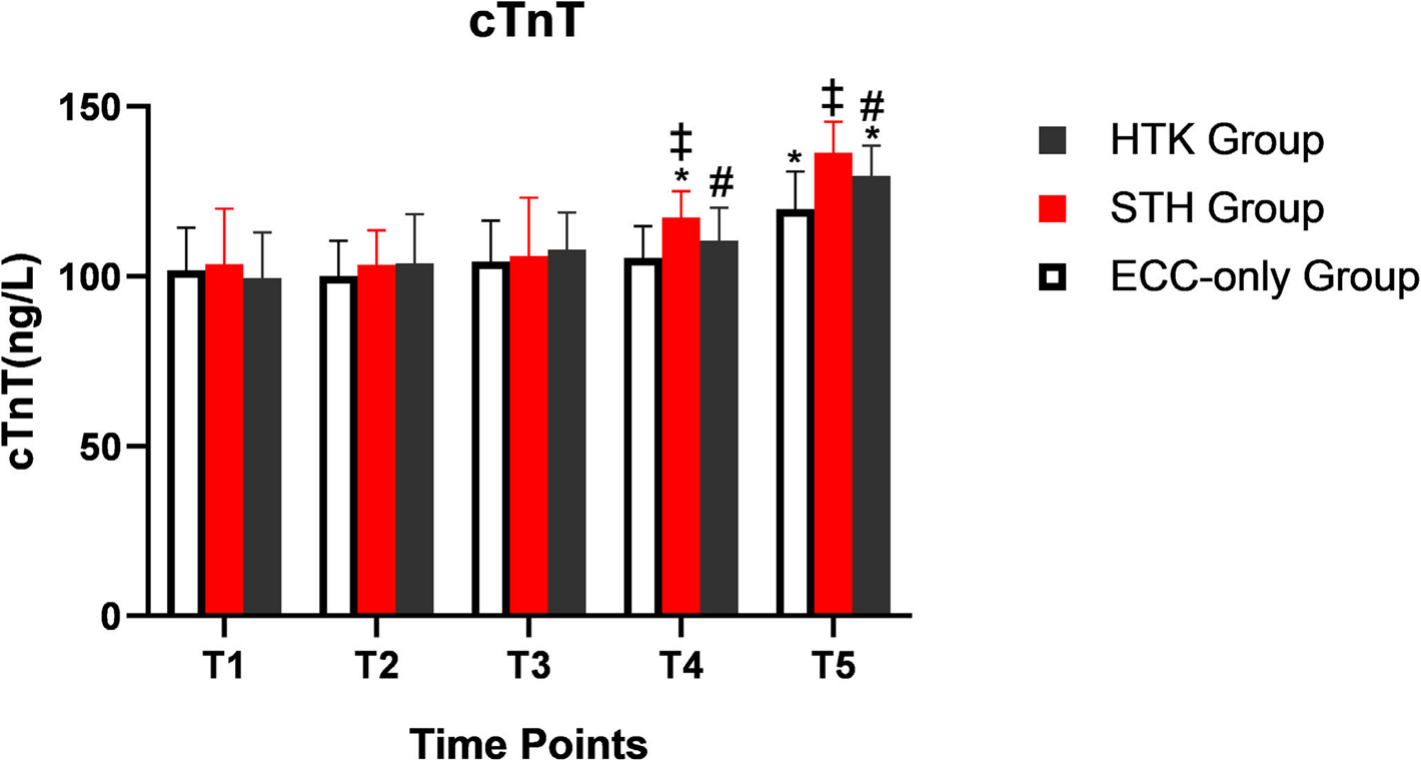
The concentration of cTnT in three different groups. Note: * *P* < 0.05, compared with the pre-ECC basis; # *P* < 0.05, compared with the STH group; ‡ *P* < 0.05, compared with the ECC-only group

There was no statistically significant difference in cTnT among the three groups at T1, T2, and T3. The cTnT level in the STH group at T4 was significantly higher than that before the start of ECC (*P* < 0.05). The cTnT level in the HTK and ECC-only groups at T5 was significantly higher than that before the start of ECC (*P* < 0.05). The concentration of cTnT was higher in the STH group than in the ECC-only group at T4 and T5 (*P* < 0.05). The concentration of cTnT was higher in the STH group than in the HTK group at T5 (*P* < 0.05) (Table 7 and Fig. 3).

### Changes in CKMB concentration

**Fig. 4:**
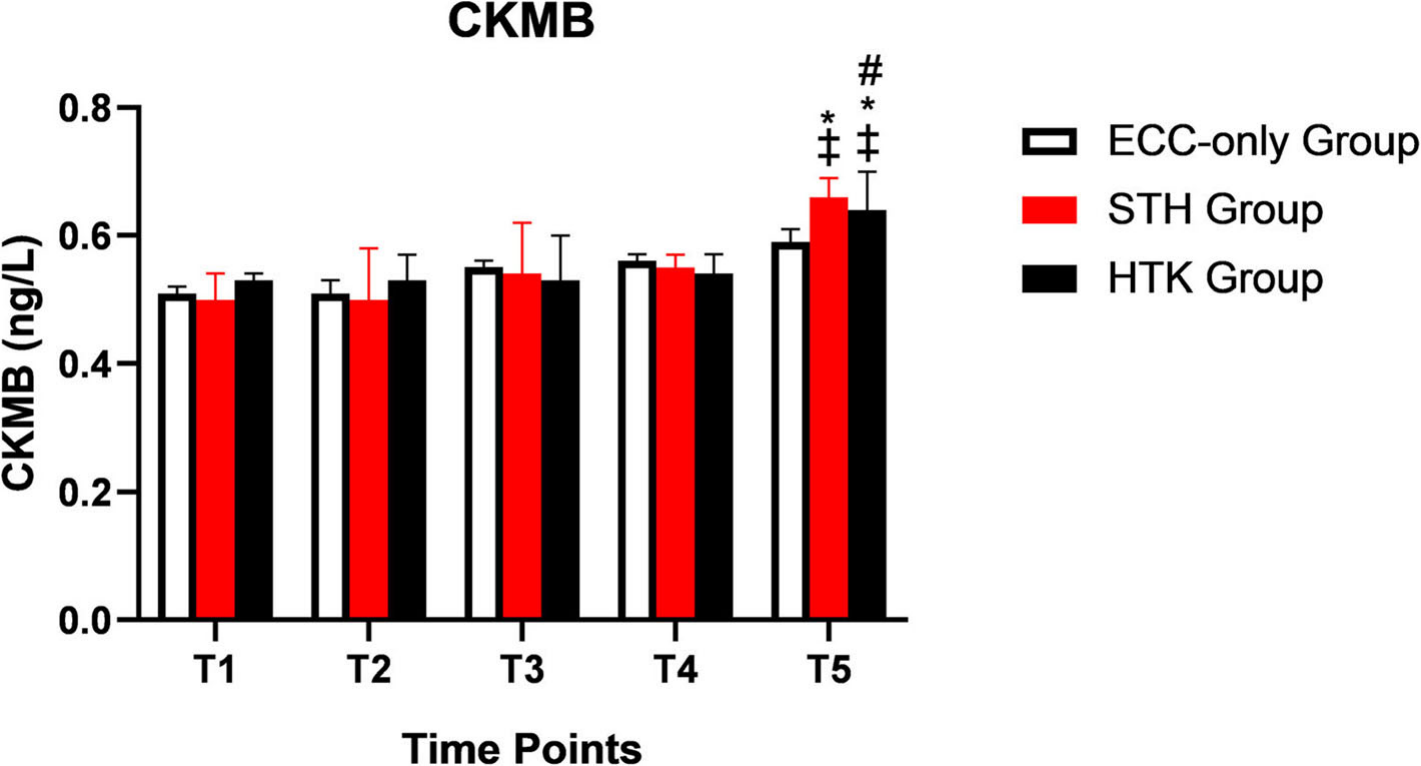
The concentration of CKMB in three different group. * *P* < 0.05, compared with the pre-ECC basis; # *P* < 0.05, compared with the STH group; ‡ *P* < 0.05, compared with the ECC-only group

There was no statistically significant difference in cTnT among the three groups at T1, T2, T3 and T5. The CKMB level in the STH and HTK groups at T5 was significantly higher than that before the start of ECC (*P* < 0.05). The concentration of CKMB was higher in the STH and HTK groups than in the ECC-only group at T5 (*P* < 0.05). The concentration of CKMB was higher in the STH group than in the HTK group at T5 (*P* < 0.05) (Table 8 and Fig. 4).

### In situ apoptosis detection in cardiomyocytes

**Fig. 5:**
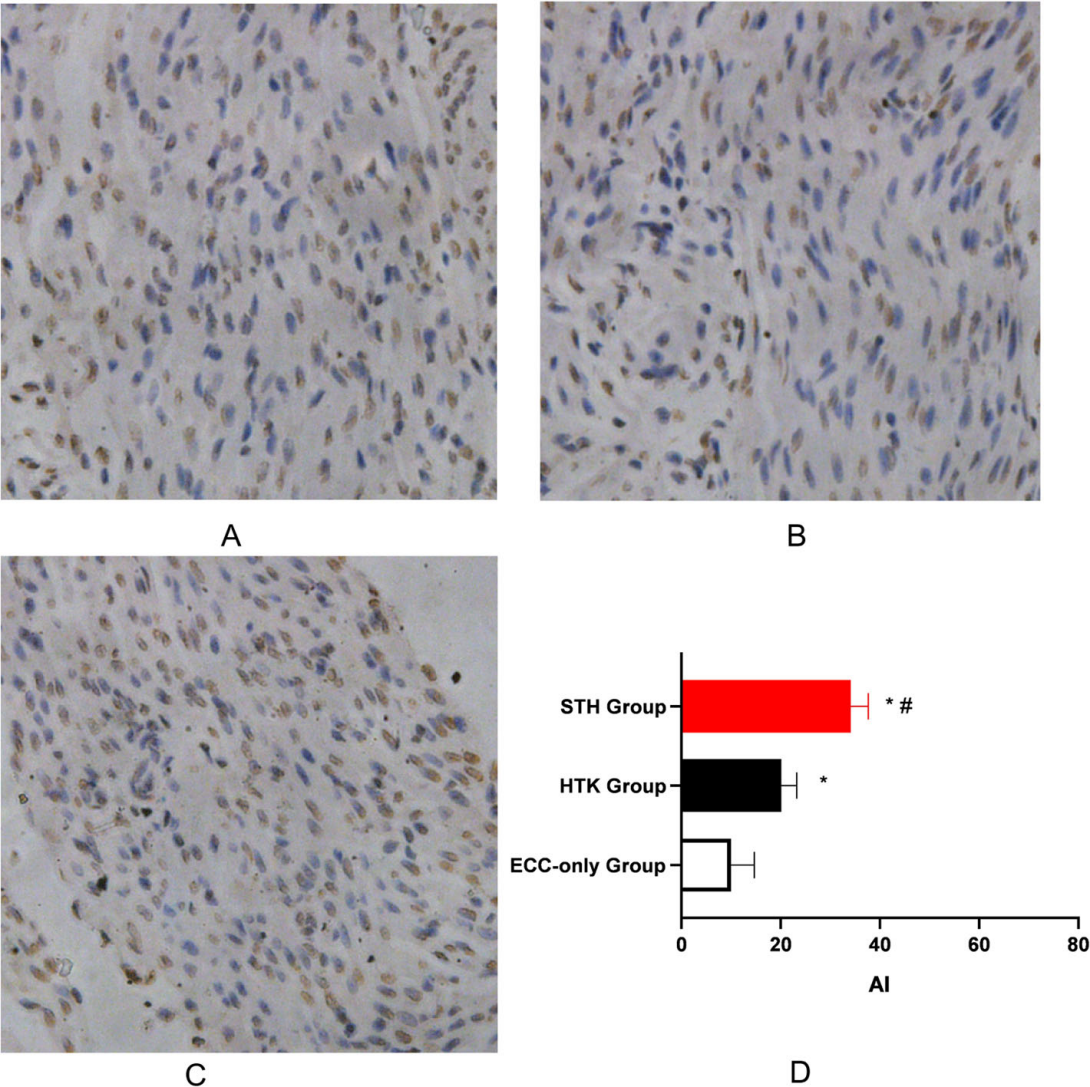
TUNEL assay to detect cell apoptosis (400×). Note: **a** Apoptosis of cardiomyocytes after ECC in the ECC-only group. **b** Apoptosis of cardiomyocytes after ECC in the HTK group. **c** Apoptosis of cardiomyocytes after ECC in the STH group. **d** Comparison of the apoptotic index of the three groups, *P < 0.05 compared with the ECC-only group; #*P* < 0.05 compared with the HTK group

The apoptotic index was calculated to be 10.2 ± 4.7 in the ECC-only group, 34.2 ± 3.7 in the STH group, and 20.2 ± 3.1 in the HTK group. The number of TUNEL-positive cells in the HTK and STH groups was significantly higher than that in the ECC-only group (*P* < 0.05). The number of TUNEL-positive cells in the STH group was significantly higher than that in the HTK group (*P* < 0.05) (Fig. 5).

## Discussion

When the heart arrests, cardiomyocytes still need to be supplied with energy to survive, but their metabolism for generating energy-producing substances is reduced: when they stop contracting, their metabolic rate is only one-tenth that of the beating state [15]. According to previous studies, St Thomas’ Hospital cardioplegic solution (STH1) and HTK preservation solution (Custodiol®) are equally safe in complex paediatric congenital heart surgery [16]. We are neutral on this point of view because fetal and infant heart development are different. There are also relatively few studies on fetal cardioprotection. In this study, cTnI, cTnT and CKMB were chosen as indicators of myocardial injury because these three myocardial biochemical markers have a good parallel relationship with myocardial damage. Myocardial injury marker levels did not differ significantly between groups before the start of ECC, but increased with the onset of ECC, indicating ischaemic and reperfusion injury. The St Thomas’ Hospital cardioplegic solution (STH1) does not provide oxygen and rich nutrients to the immature myocardium [17]. In this study, the levels of cTnI, cTnT and CKMB in the HTK group were lower than those in the STH group after ECC, and the number of apoptotic cells was lower.

HTK preservation solution is currently considered to be the best cardioplegic solution. This study showed that HTK preservation solution (Custodiol®) was significantly more protective of immature myocardium than St Thomas’ Hospital cardioplegic solution (STH1), and significantly reduced ischaemia and ischaemia-reperfusion injury to immature myocardium due to hypoxic ischaemia. The reasons for this include the fact that the K+ concentration in HTK preservation solution is only 9 mmol/L, which is significantly lower than that in St Thomas’ Hospital cardioplegic solution (STH1), thus avoiding high K + -induced endothelial damage in the coronary arteries and facilitating the maintenance of capillary and extracellular interstitial homeostasis in tissues and organs [18, 19]. In addition, many studies have shown that a single infusion of HTK preservation solution (Custodiol®) can effectively arrest the heart for 180 min. Because of this feature, HTK preservation solution (Custodiol®) protects the myocardial cells better without limiting the duration of the procedure [20]. Endogenous nucleic acid endonucleases cleave DNA at nucleosomes during apoptosis. The TUNEL method actually stains the severed ends of the cut DNA [21]. We observe apoptosis by looking at DNA breaks in the nucleus. In this experiment, we found that the number of TUNEL-positive cells was higher in the STH group than in the HTK group. This also indicates that HTK preservation solution (Custodiol®) provides greater protection to cardiomyocytes than St Thomas’ Hospital cardioplegic solution (STH1). However, the number of TUNEL-positive cells in HTK group was still higher than in ECC-only group, indicating that the protection of cardiomyocytes by HTK preservation solution (Custodiol®) was also limited.

Our study has some limitations. For example, it is difficult to obtain laboratory animals that are suitable for the conditions, resulting in a small sample size. In immature myocardium, damage due to the ECC often peaks at 6–8 h postoperatively, and the degree of apoptosis at 120 min postoperatively is not necessarily representative of apoptosis at 6–8 h postoperatively. False-positive results can also occur in TUNEL reactions. We were unable to find a suitable ultrasound instrument to measure the relevant data on cardiac function in real time. The use of data such as heart rate and mean arterial pressure alone does not accurately reflect cardiac function. Moreover, there are genetic differences between human and sheep immature cardiomyocytes. Therefore, more experimental evidence is needed to fully evaluate the cardioplegic solutions effects on the fetal myocardium.

## Conclusion

Our study shows that in an extracorporeal circulation model in fetal sheep, HTK preservation solution (Custodiol®) has a significant advantage over St Thomas’ Hospital cardioplegic solution (STH1) in reducing the release of markers of cardiomyocyte injury and a lower number of apoptotic cells. Therefore, HTK preservation solution (Custodiol®) offered greater protection to cardiomyocytes than St Thomas’ Hospital cardioplegic solution (STH1). We found that fetal sheeps using only ECC had an advantage in all indicators. The number of TUNEL-positive cells in the HTK group was higher than that in the ECC-only group, indicating that the protective effect was still limited. It provides a theoretical basis for future fetal heart surgery with ECC only.

## Data Availability

All data generated or analysed during this study are included in this published article [and its supplementary information files].

## Abbreviations

CKMB: Creatine kinase–muscle band
ECC: Extracorporeal circulation
cTnI: Troponin I
cTnT: Troponin T
HTK: HTK preservation solution (Custodiol®)
STH: St Thomas’ Hospital cardioplegic solution (STH1)

## References

[1] Roger VL, Go AS, Lloyd-Jones DM, Adams RJ, Berry JD, Brown TM, Carnethon MR, Dai S, de Simone G, Ford ES, Fox CS, Fullerton HJ, Gillespie C, Greenlund KJ, Hailpern SM, Heit JA, Ho PM, Howard VJ, Kissela BM, Kittner SJ, Lackland DT, Lichtman JH, Lisabeth LD, Makuc DM, Marcus GM, Marelli A, Matchar DB, MM MD, Meigs JB, Moy CS, Mozaffarian D, Mussolino ME, Nichol G, Paynter NP, Rosamond WD, Sorlie PD, Stafford RS, Turan TN, Turner MB, Wong ND, Wylie-Rosett J, American Heart Association Statistics Committee and Stroke Statistics Subcommittee (2011). Heart disease and stroke statistics--2011 update: a report from the American Heart Association. Circulation.

[2] Becker S, Hofbeck M, Kendziorra H, Wallwiener D, Mielke G (2004). Double-chamber right ventricle associated with severe fetal cardiac failure. Ultrasound Obstet Gynecol.

[3] Huhta JC (2004). Guidelines for the evaluation of heart failure in the fetus with or without hydrops. Pediatr Cardiol.

[4] Sklansky M (2003). New dimensions and directions in fetal cardiology. Curr Opin Pediatr.

[5] Sun R, Liu M, Lu L, Zheng Y, Zhang P (2015). Congenital heart disease: causes, diagnosis, symptoms, and treatments. Cell Biochem Biophys.

[6] Dolkavt LA, Reimers FT (1991). Transvaginal fetal echocardiography in early pregnancy: normative data. Am J Obstet Gynecol.

[7] Arzt W, Tulzer G (2011). Fetal surgery for cardiac lesions. Prenat Diagn.

[8] Medikonda R, Ong CS, Wadia R, Goswami D, Schwartz J, Wolff L, Hibino N, Vricella L, Nyhan D, Barodka V, Steppan J (2019). Trends and updates on cardiopulmonary bypass setup in pediatric cardiac surgery. J Cardiothorac Vasc Anesth.

[9] Reddy VM, Liddicoat JR, Klein JR (1996). Long-term outcome after fetal cardiac bypass:fetal survival to full term and organ abnormalities [J]. J Thorac Cardiovasc Surg.

[10] Duffy JY, Petrucci O, Baker RS, Lam CT, Reed CA, Everman DJ, Eghtesady P (2011). Myocardial function after fetal cardiac bypass in an ovine model. J Thorac Cardiovasc Surg.

[11] Reddy VM, Liddicoat JR, Klein JR, McElhinney DB, Wampler RK, Hanley FL (1996). Fetal cardiac bypass using an inline axial flow pump to minimize extracorporeal surface and avoid priming volume. Ann Thorac Surg.

[12] Oliveira MS, Floriano EM, Mazin SC (2011). Ischemic myocardial injuries after cardiac malformation repair in infants may be associated with oxidative stress mechanisms. [J]. Cardiovasc Pathol.

[13] Yamamoto F (2010). Metabolic characteristics of immature myocardium. [J]. Gen Thorac Cardiovasc Surg.

[14] Chang JP, Chen MC, Lin WY, Liu WH, Chen CJ, Chen YL, Pan KL, Tsai TH, Chang HW (2011). DNA repair in TUNEL-positive atrial cardiomyocytes of mitral and tricuspid valve diseases: potential mechanism for preserving cardiomyocytes. Int J Cardiol.

[15] Landymore R, Murphy JT, Hall R (1996). Randomized trial comparing intermittent antegrade warm blood cardioplegia with multidose cold blood cardioplegia for coronary artery bypass [J]. Eur J Cardiothorac Surg.

[16] Wang ZH, An Y, Du MC, Qin TJ, Liu YB, Xu HZ, Yang LQ (2017). Clinical assessment of histidine-tryptophan-ketoglutarate solution and modified St. Thomas’ solution in pediatric cardiac surgery of tetralogy of fallot. Artif Organs.

[17] Gottlieb RA, Gruol DL, Zhu JY, Engler RL (1996). Preconditioning rabbit cardiomyocytes: role of pH, vacuolar proton ATPase, and apoptosis. J Clin Invest.

[18] Mohara J, Tsutsumi H, Takeyoshi I (2002). The opti⁃mal pressure forinitial flush with UW solution in heart procure⁃ment [J]. J Heart Lung Transplant.

[19] Zaman MJ, Vrotsou K, Chu GS (2011). A high incidental rise in cardiac troponin I carries a higher ortality risk in older patients than in those with a diagnosed acute coronary syndrome [J]. Age Ageing.

[20] Prathanee S, Kuptanond C, Intanoo W, Wongbhudha C, Karunasumaeta C (2015). Custodial-HTK solution for myocardial protection in CABG patients. J Med Assoc Thail.

[21] Kyrylkova K, Kyryachenko S, Leid M, Kioussi C (2012). Detection of apoptosis by TUNEL assay. Methods Mol Biol.

